# EFFECT OF SUPPLEMENTATION WITH PROBIOTICS ON COLONIC ANASTOMOSES IN RATS: MORPHOLOGICAL AND TENSIOMETRIC STUDY

**DOI:** 10.1590/0102-672020200004e1550

**Published:** 2021-01-25

**Authors:** Tiago Jacometo Coelho de CASTILHO, Gustavo Henrique Doná Rodrigues de ALMEIDA, Eneri Vieira de S. L. MELLO, Antônio Carlos L. CAMPOS

**Affiliations:** 1 Postgraduate Program in Surgical Clinic, Health Sciences Sector, Federal University of Paraná, Curitiba, PR, Brazil; 2 Animal Histotechnical Laboratory, Department of Morphophysiological Sciences, State University of Maringá, Maringá, PR, Brazil

**Keywords:** Probiotic, Wound healing, Colonic anastomosis, Tensiometers, Collagen densitometry, Rats, Probiótico, Cicatrização ferida, Anastomose colônica, Tensiômetro, Densitometria do colágeno, Ratos

## Abstract

**Background:**

The use of probiotics positively modifies the composition and function of intestinal flora, improving the quality of intestinal anastomosis.

**Aim:**

To evaluate the impact of probiotic use on intestinal anastomosis of rats.

**Method:**

Thirty-six adult male Wistar rats (*Rattus norvegicus albinus*, Rodentia Mammalia) were used, with body weight ranging from 220-320 g. The animals were housed and acclimated individually in boxes receiving water and ration ad libitum. After initial acclimatization, the control group received perioperative ration ad libitum for 12 days (seven preoperatively and five postoperatively) associated with the maltodextrin formula at a dose of 250 mg/day in isocaloric and isovolumetric form. Likewise, the probiotic group received oral supplementation of probiotics dose of 250 mg/day, associated with isocaloric and isovolumetric diet. The probiotic chosen for this study was composed of strains (doses 1x10^9^ CFU/g)^12^ *Lactobacillus paracasei* LPC-37, *Bifidobacterium lactis* HN0019, *Lactobacillus rhamnosus* HN001 and *Lactobacillus acidophilus* NCFM. Probiotics or placebo were administered orally with the aid of a dosimeter spatula. Both groups underwent two colostomies, one in the right colon and the second in rectosigmoid, followed by reanastomosis with eight separate 6-0 mononylon stitches. The sacrifice took place on the fifth day. The parameters evaluated included tensile strength, histology and collagen densitometry.

**Results:**

The rate of intestinal fistula for the control and probiotic groups were, respectively, 22.22% and 11.11% (p*=*0.6581*)*.Perioperative supplementation with probiotics increased collagen deposition of types I and III (p<0.0001), improved maximum traction force and maximum rupture force, p=0.0250 and p=0.0116 respectively, fibrosis area (p<0.0001), and area of the inflammatory infiltrate (p=0.0115).

**Conclusions:**

The use of probiotics had a positive impact on the quality of intestinal anastomosis**.**

## RESUMO

**Racional:**

A utilização de probióticos modifica positivamente a composição e função da flora intestinal melhorando a qualidade da anastomose intestinal.

**Objetivo:**

Avaliar o impacto da utilização de probióticos na anastomose intestinal de ratos.

**Método:**

Foram utilizados 36 ratos Wistar (*Rattus norvegicus albinus,* Rodentia Mammalia), machos adultos, com peso corporal variando entre 220 e 320 g. Os animais foram alojados e aclimatados individualmente em caixas recebendo água e ração ad libitum. Após aclimatação inicial, o grupo controle recebeu perioperatoriamente ração ad libitum por 12 dias (sete no pré-operatório e cinco no pós-operatório) associado à fórmula de maltodextrina na dose de 250 mg/dia de forma isocalórica e isovolumétrica. Na semana que precedeu o procedimento cirúrgico (período de sete dias) e no pós-operatório (por cinco dias), os ratos do grupo estudo receberam suplementação via oral de probióticos dose de 250 mg/dia, associado à dieta isocalórica e isovolumétrica. O probiótico utilizado era composto pelas cepas (doses 1x10^9^ UFC/g)^12^ *Lactobacillus paracasei* LPC-37, *Bifidobacterium lactis* HN0019, *Lactobacillus rhamnosus* HN001 e *Lactobacillus acidophilus* NCFM. A administração de probiótico ou placebo foi realizada via oral, com auxílio de espátula com dosímetro. Os dois grupos foram submetidos à duas colostomias, uma em cólon direito e outra em retossigmóide, seguido de reanastomose com oito pontos separados de mononylon 6-0. O sacrifício ocorreu no quinto dia. Os parâmetros avaliados incluíram força tênsil, histologia e densitometria do colágeno.

**Resultados:**

A taxa de fístula intestinal para os grupos controle e probiótico foram, respectivamente, 22,22% e 11,11% (p=0.6581*)*. A suplementação peroperatória com probióticos aumentou a deposição de colágeno dos tipos I e III (p<0.0001), melhorou a força máxima de tração e força máxima de ruptura, p*=*0,0250 e p*=* 0,0116 respectivamente, área de fibrose (p<0.0001), e área do infiltrado inflamatório (p=0.0115).

**Conclusões:**

A utilização de probióticos impactou positivamente na qualidade da anastomose intestinal.

## INTRODUCTION

Probiotics, in a broad definition, can be considered dietary substances that promote changes in the composition and/or activity of the gastrointestinal microbiota that end up conferring benefits to the health of the host. In order to understand the role of probiotics in homeostasis and health, it is important to note that the human gastrointestinal tract hosts more than 500 species of bacteria, totaling a weight of approximately 1 kg, with a proportion of 10 bacteria for each human cell. The bacterial genome can be present in the proportion of 100:1, and more than 10% of the daily energy required by an individual can be derived from bacterial fermentation^4^ ^,^ ^7^ ^,^ ^11^ ^,^ ^18^.

Strictly speaking, they are live bacteria or specific yeasts that meet criteria: non-pathogenicity, ability to antagonize pathogenic bacteria, resistance to gastric acidity or lysis by bile, adhesion to the epithelium, being a source of modulation of host immunity and having the ability to remain stable during processing and storage^3^ ^,^ ^6^.

It was believed that the benefits of probiotics came mainly from promoting the balance of the intestinal microbiota; however, there is increasing evidence that they also play an important immunomodulatory function^3^ ^,^ ^30^.

Initially, without the presence of any type of bacteria in our gastrointestinal tract - and therefore, without antigens for the development of tolerance and immunity -, our defense system begins to develop in the lymphoid tissue associated with the gastrointestinal tract, whose exposure early to microbial antigens promotes their colonization to the point that at four years of age we already have a stable microbiome configuration^3^.

In this process, we have the intraluminal conversion of components of our diet through the already colonized gastrointestinal tract. The diet modulates the microbiota, the changes being verified by experimental models in rats. An example of this is the fact that a diet rich in fats increases the proportion of *Clostridium ramosum* and a reduction in the proportion of bacterioids^9^ ^,^ ^27^. The levels of serotonin, a neurotransmitter derived from tryptophan, were modulated by the colonization of *Bifidobacterium infantis*, which increased their serum bioavailability and frontal cortex in rats^2^ ^,^ ^5^. Other important actions described are that probiotics and their metabolites reduce the secretion of the autoinducer-2 immunomodulatory molecule by pathogenic *E.coli*, which ultimately functions as a mediator of bacterial adhesion to intestinal cells, thus not allowing enteroinvasion^13^.

The beneficial effects and clinical applications of probiotics are the most diverse and are due to the most different mechanisms, among which we can highlight: reduction of intraluminal pH, secretion of bactericins and defensins. This set of probiotic actions promotes antagonistic activity with enteropathogens and uropathogens^10^. The barrier function of the intestinal mucosa is enhanced by phosphorylation of actinin and ocludine at the narrow junctions and, also, by inhibiting apoptosis induced by cytokines^28^. Probiotics also induce the secretion of HeatShock Protein-70 (HSP-70), which are proteins present in the cytosol, which act as molecular guides in preventing protein aggregation and helping in the processing of antigens and their presentation^15^ ^,^ ^25^. Animal models demonstrated the protection exercised in spontaneous and chemically induced colitis through the “downregulation” of pro-inflammatory cytokines, related to specific strains. Other mechanisms identified were the reduction of CD4+ lymphocytes from the lamina propria and intraepithelial lymphocytes, inhibition of necrosis factor alpha (TNF-alpha), production of type 1 monocyte chemoattractive protein (MCP-1), in addition to increased production of interleukin-10 (IL-10)^3^ ^,^ ^17^.

Although probiotics perform functions ranging from immunomodulation to the bioavailability of neurotransmitters to the central nervous system, their effects on tissue repair in colonic anastomoses are still unknown.

The aim of this study was to evaluate the effect of perioperative probiotic supplementation on the healing process of colonic anastomoses in rats.

## METHODS

Thirty-six Wistar rats (*Rattus norvegicus albinus*, Rodentia Mammalia), adult males, with body weight varying between 220-320 g, from the Vivarium of the State University of Maringá, Maringá, PR, Brazil, were used. The animals were housed and acclimated individually in boxes, receiving water and food ad libitum.

After initial acclimatization, the control group received food with ad libitum food for 14 days associated with the formula of maltodextrin (n=18) at a dose of 250 m/day in an isocaloric and isovolumetric way. The probiotic study group (n=18) received ad libitum food for two weeks. In the week preceding the surgical procedure (seven-day period) and in the postoperative period (five-day period), the rats of the study group received oral supplementation of probiotics at a dose of 250 mg/day, associated with the isocaloric and isovolumetric diet.

The probiotic chosen for this study was composed of the strains *Lactobacillus paracasei* LPC-37, *Bifidobacterium lactis* HN0019, *Lactobacillus rhamnosus* HN001 and *Lactobacillus acidophilus* NCFM (doses 1x109 UFC/g)^12^.

The administration of probiotic or placebo was performed orally with the aid of a spatula with dosimeter containing the appropriate dose of the probiotic or maltodextrin administered directly (oral). Both groups underwent two colostomies, one in the right colon and the other in rectosigmoid, followed by reanastomosis with eight separate 6-0 mononylon stitches. The sacrifice took place on the fifth day. The parameters evaluated include tensile strength, collagen histology and densitometry.

### Statistical analysis

Statistical analysis was performed using the T-Student and Mann-Whitney test with a 95% confidence interval (CI).

## RESULTS

There was intestinal fistula in the rectosigmoid anastomosis in four animals (22.22%) in the control group and two (11.11%) in the probiotic group. The animals that presented fistula died.

### Tensiometric test

**FIGURE 1:**
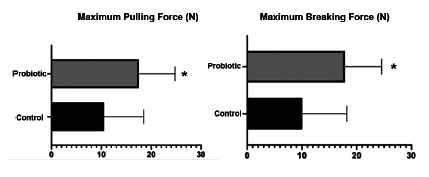
Comparison between maximum pulling force (FMT) and maximum breaking force (FMR) between the control and probiotic groups. Data presented by mean±standard error. Mann-Whitney test *p<0.05 compared with the control group.

To assess tensile strength, a computerized mechanical testing machine of the brand EMIC model DL 1000 (EMIC, São José dos Pinhais - PR) was used. The value of the maximum traction force (FMT) in the control group was 1.2±0.2 N and in the probiotic group it was 1.5±0.3 N, p=0.0250 95.27% CI. The maximum breaking strength (FMR) of the control and probiotic groups were, respectively, 1.1±0.2 N and 1.4±0.3 N, p=0.0116 95.27% CI. Thus, it was possible to verify the positive impact on the use of the probiotic on the anastomosis tensile strength (Figure 1).

### Histological evaluation and densitometry of collagen

The material was fixed in 10% formalin and processed for histopathological evaluation, with the following parameters being analyzed: area of inflammatory process, inflammatory infiltrate, fibrosis, vascular congestion, granulation tissue and edema, using H&E staining. The cuts were standardized at six micrometers thick. The analysis of collagen densitometry was obtained by staining Picrosirius Red 3FBA (PSR 3FBA) with automated reading by the GraphPad Prism 5 system.

**FIGURE 2:**
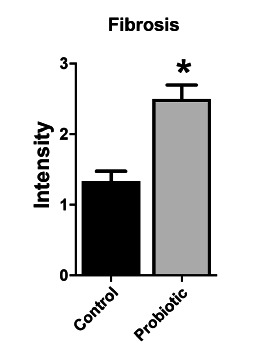
Comparison between the histopathological indexes (IH) of fibrosis between the control and probiotic groups. Data presented by mean±standard error. Student T test. *p<0.05 compared to the control group

**FIGURE 3:**
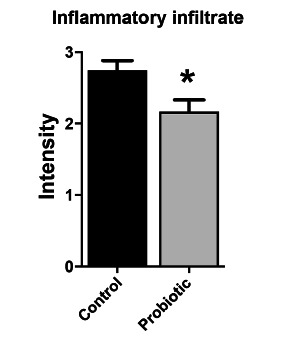
Comparison between the histological indices (HI) of inflammatory infiltrate between the control and probiotic groups. Data presented by mean±standard error. Student T test. *p<0.05 compared to the control group.

In the H&E analysis, there was no difference between the groups in relation to edema, vascular congestion and granulation tissue (p>0.05); however, there was a significant difference (p<0.05) for fibrosis and inflammatory infiltrate, as well as for the inflammatory process area (Figures 2 and 3).

**FIGURE 4:**
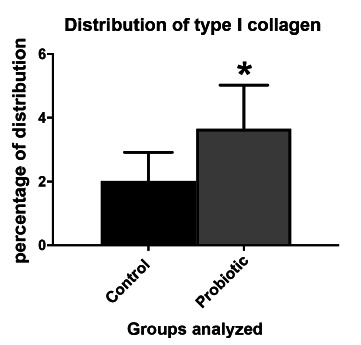
Distribution of type I collagen (COL I) between control and probiotic groups. Data presented by mean±standard error. Student T test. *p<0.05 compared to the control group.

**FIGURE 5:**
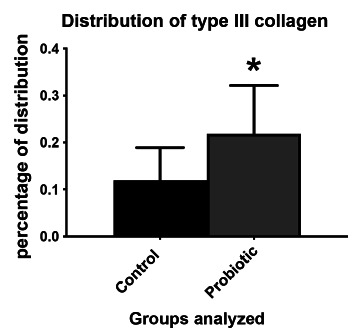
Distribution of type III collagen (COL III) between control and probiotic groups. Data presented by mean±standard error. Student T test. *p<0.05 compared to the control group.

In the evaluation of PSR, the presence of collagen types I and III was significantly higher (p<0.0001) in the probiotic group (Figures 4 and 5).

## DISCUSSION

The intestinal mucosa is a complex and dynamic system that functions as a semipermeable, allowing the passage of nutrients and macromolecules necessary for growth and development while protecting the bloodstream from potentially invasive microorganisms^19^. These basic functions occur in an environment inhabited by billions of commensal microorganisms from the three groups of living beings: *Bacteria* (Firmicutes, Bacteroidetes, Proteobacteria and Actinobacteria), which constitute about 96% of the intestinal microbiota, *Archea* and Eukarya, as well as viral particles^1,7.20^.

The resection of a gastrointestinal segment is a common procedure for surgeons for a variety of reasons: gastroplasty, neoplasms, diverticular disease, inflammatory diseases, obstruction, etc. Considering intestinal anastomoses, all of its layers (from mucosa to serosa) will be sectioned and the tissue re-approximated with staplers and sutures. Thus, we will have “foreign bodies” in the healing line that act as antigens for our defense system.

Despite all the progress in terms of the means and materials to perform an operation (laparoscopy, robotics, etc), the surgeon’s dexterity to perform the procedure with an appropriate technique, the rates of anastomotic fistulas have remained the same for over 60 years^23^ . In this sense, we seek to verify the possible contributing factors to these stable rates of complications. Both the species and the bacterial phenotype seem to be involved primarily in the complication.

The results obtained in the present study suggest the presence of a more “organized” healing process with adequate intensity given the greater resistance in the tests of traction and collagen deposition obtained. In this same sense, it must be considered that the process was more effective taking into account that the probiotic group had a smaller area of ​​inflammatory infiltrate than the control group. Among the processes involved is the expression of Toll-like receptors (TRLs) expressed by the commensal microbiota responsible for inducing chemokine with chemotactic activity and modulating the function of dendritic cells and T lymphocytes to promote and modulate wound healing^31^.

In a study conducted by Olivas et al.^16^, it was found that when subtypes with enteropathogenic characteristics of *Pseudomonas aeruginosa* were present at the anastomosis site, the fistula was present. When evaluating the mechanisms by which strains of *Escherichia, Enterococcus* and other groups of bacteria contribute to the formation of fistulas, increases in pore formation were found through H&E, increased expression of the AIDA-I22 adhesion protein, direct cytotoxicity and through the release of tumor necrosis factor^8^ ^,^ ^24^, high motility and especially the increase in proteolytic activity of gelatinase, causing the degradation of the collagen matrix at the anastomosis site^26^. It has been observed that the intestinal anastomoses of animals treated with antibiotics considered “germ-free”, when submitted to tensiometric tests, have a lower explosive strength or “burststregth” than animals not treated with antibiotics^12^ ^,^ ^14^ ^,^ ^29^, suggesting that the imbalance in the usual microbiota reduces the quality of the anastomosis. On the other hand, in other animal models that made empirical use of antibiotics, these proved to be useful in preventing fistulas when pathogenic bacteria or underlying diseases were present^21^.

## CONCLUSIONS

Perioperative supplementation with probiotics was positively associated with increased deposition of types I and III collagen, increased tensile strength and area of ​​fibrosis, even though there was initially an increase in the inflammatory infiltrate.
